# Mechanical, Dielectric, and Spectroscopic Characteristics of “Micro/Nanocellulose + Oxide” Composites

**DOI:** 10.1186/s11671-017-1862-x

**Published:** 2017-02-08

**Authors:** Maksym Nedielko, Smail Hamamda, Olexander Alekseev, Vitalii Chornii, Mykola Dashevskii, Maksym Lazarenko, Kostiantyn Kovalov, Sergii G. Nedilko, Sergii Tkachov, Sergiy Revo, Vasyl Scherbatskyi

**Affiliations:** 1 O. Paton Electric Welding Institute of NASU, Bozhenko str. 11, 03680 Kyiv, Ukraine; 2 University Frères Mentouri, B.P. 325 Route Ain El Bey, 25017 Constantine, Algeria; 3 Taras Shevchenko National University of Kyiv, Volodymyrska str. 64/13, 01601 Kyiv, Ukraine

**Keywords:** Cellulose, Micro/nanosize, Morphology, Oxide, Composite, Luminescence

## Abstract

The set of composite materials that consist of micro/nanocellulose and complex K_2_Eu(MoO_4_)(PO_4_) luminescent oxide particles was prepared. The composites were studied by means of scanning electron microscopy, XRD analysis, dilatometry, differential scanning calorimetry and thermogravimetric analysis, and dielectric and luminescence spectroscopy.

Dependencies of density, crystallinity, relative extension, thermal extension coefficient, dielectric relaxation parameters, intensity and shape of photoluminescence bands on temperature, and content of oxide component were studied. The structure of the composite without oxide is formed by grains of nearly 5–50 μm in size (crystallinity is about ~56%). Structure of the micro/nanocellulose samples which contain oxide particles is similar, but the cellulose grains are deformed by oxide particles. Dependencies of the abovementioned properties on temperature and oxide content were analyzed together with data on the size distribution of oxide particles for the samples for various oxide and molecules of water concentrations.

## Background

Due to its multifunctionality, low toxicity, biodegradability, and abundance (annual production in the biosphere is of about 90 billion tons), cellulose is important for the production of advanced materials, biofuels, biochemicals, etc. [1]. The range of cellulose’s promise spreads from “paper electronics” (“paper transistor” on a carbon nanotube or organic LED), based on luminescent cellulose [2–4], to forensic examination and eco-friendly sorbents suitable for sorption and stabilization of a wide range of various types of materials [5]. This variety of possible applications is determined by the porous, micro-, and nanostructured morphology of the cellulose host and by the unique nature of its interaction with other chemical compounds [6–8].

Cellulose nanomaterials have also strong promise for use as reinforcements in polymer matrix composites due to their light weight, high tensile strength and modulus, and comparably low cost [9]. Now, polymers reinforced with natural “biocomposites” attracted industrial application not only in the automotive or building sectors but also in the wide area of goods [9]. The potential market for nanocellulose is very extensive. It can be conditionally divided on three areas of applications [1]: novel and emerging, low volume, and, finally, high-volume applications. The automobile body, automobile interior, cement, hygiene and absorbent products, packaging coating, packaging filler, plastic film replacement, replacement plastic packaging, and textiles for clothing are related to the area of high-volume applications [1]. It is easy to see that they are close to the traditional applications of macrocellulose. Aerospace interiors, aerogels for the oil and gas industry, aerospace structure, insulation, architectural paint, OEM application paint, special purpose paint, and wallboard facing are those which are capable of changing the mentioned industry trends, and they constitute low-volume applications of nanocellulose [1]. And finally, medical sensors, environmental, industrial, reinforcement fiber for construction, water filtration, miscellaneous viscosity modifiers, purification, cosmetics, organic light-emitting diodes (LEDs), flexible electronics, photovoltaics, recyclable electronics, 3D printing, and photonic films should be related with novel and emerging applications [1].

It is common knowledge that cellulose is a very effective sorbent, too. Really, synthetic sorbents based on polyethylene, propylene, and other polymers show better performance, but they are not eco-friendly enough in comparison to cellulose [10, 11].

Cellulose-oxide micro/nanocomposite materials that consist of host micro/nanocellulose and micro/nanoparticles of inorganic oxides (COM/NC) are among the advanced directions of possible high-tech micro/nanocellulose application (see, e.g., [12]). It concerns particularly luminescent COM/NC materials [13–15]. Some of the luminescent oxide nanomaterials containing luminescent lanthanide ions have been studied by us in detail (see [16–21] and references therein) that enabled us now to select some of them for incorporation into the micro/nanocellulose host.

The aim of this work was to fabricate using cool-pressing procedure the set of cellulose-oxide micro/nanocomposite materials and to study their physical properties. Properties of the micro/nanocellulose sufficiently depend on its fabrication procedure, methods of its functionalization, and modification. Therefore, micro/nanocellulose materials made by us have been widely characterized. So, their mechanical, thermal, thermomechanical, dielectrical, optical, etc. characteristics were found. The made COM/NC were characterized for surface topography and morphology by scanning electron microscopy (SEM), X-ray diffractometry (XRD), thermal stability through thermal properties measurements by thermogravimetric analysis (TGA and differential scanning calorimetry (DSC)), and luminescence spectroscopy. The crystallinity index, dielectric permittivity, shape, and size of the composite component were evaluated as suitable for different applications.

## Methods

### The Samples

Chemically pure microcrystalline cellulose tablets manufactured at ANCYR-B (Ukraine) were used as starting material for preparation of the composite samples under study. (Starting cellulose materials are marked below as SC.) First, cellulose tablets were grinded and dispersed using a high-speed rotation mill. Then, some part of the resulting powder was mixed with a certain amount of the previously made nanosized oxide powder. The mix of ~0.5-g mass was dissolved in 50 ml of high-purity ethanol and undergone to ultrasonic treatment (frequency, *f* = 4.2 kHz, time of the treatment, *t* = 20 min). After that, obtained suspension stood for 30 min and the obtained precipitate was filtered via filter paper. The prepared powder was dried at ambient air conditions and room temperature (RT) followed by compression at high pressure of 1.8 × 10^4^ kPa/m^2^. Finally, the made durable cellulose-oxide micro/nanocomposite (COM/NC) samples were in disc form of average diameter near 10.3 mm. The thickness of the discs was in the range 0.78–1.14 mm that depends on the sample composition.

Doped with luminescent europium three charged ions, the Eu^3+^ complex oxide compound, K_2_Eu(PO_4_)(MoO_4_), was used as the oxide component of the composites. These powdered micro/nanosized compounds with various content of the Eu^3+^ ions have been synthesized and studied previously [22–24]. These compounds revealed intensive structured photoluminescence (PL) related to the intrinsic radiation transition in the *f-f* electronic shell of the Eu^3+^ ions.

Three identical sets of the COM/NC samples which differ by content of complex oxide, C (wt%): C = 0% (un-doped micro/nanocrystalline cellulose, M/NC, marked as C0), 0.22% (marked as C1), 2.2% (C2), and 18.2% (C3), were prepared for further studies. Various samples of each set were used for derivatography, mechanical and thermomechanical, dielectric characteristics, and luminescence measurements. The samples from one of the set were placed between two stainless steel plates, and the last ones were slightly pressed. These samples were used for the dielectric studies.

### Equipment

Characterization of the sample topology was performed by means of the scanning electron microscopy (SEM). Scanning electron microscope JAMP-9500F Field Emission Auger Microprobe (JEOL, USA) equipped with X-ray microanalyzer INCA PentaFETx3 (Oxford instruments) was used for SEM measurements. Besides the SEM imaging, microelement analysis of various areas of the samples was also performed using the same microscope.

The X-ray diffraction (XRD) patterns were collected using a conventional powder diffractometer DRON-3M equipped with BSV-28 tube (*λ*_rad_ = 1.54178 Å) and operating in Bragg-Brentano (*θ*/2*θ*) geometry. The XRD patterns were obtained in the (2*θ*) diffraction angle range 10–70° at 0.1° step.

The Netzsch 402C dilatometer (NETZSCH, Selb, Germany) with 3% accuracy was used to study thermoexpansion characteristics and a coefficient of thermal expansion was measured in the temperature range of 25 to 110 °C. The dilatometric measurements were carried out along the normal of the disc base. The heating rate used during measurements was 10 °C/min.

Differential scanning calorimetry (DSC) and thermogravimetric analysis (TGA) testing were made using the Jupiter STA 449 F3 calorimeter by Netzsch (NETZSCH, Selb, Germany). The same heating rate was used as in the dilatometric measurements.

The sample for dielectric studies was placed into a thermostatic four-electrode cell that enabled monitoring the sample thickness by means of an additional air capacitor. Measurements of the capacity and dielectric loss index of the mentioned cell were carried out at four fixed frequencies, *f* = 5, 10, 20, and 50 kHz in the temperature range −180 to 130 °C. The automatic homemade equipment on the base of the P5083 alternate current bridge was used for the abovenoted measurements.

The PL and the PL excitation spectra were measured using the spectrometric equipment SDL-2M. The PL emission spectra were analyzed using a single-grating (1200 grooves/mm) monochromator MDR-23 (linear dispersion 0.5 mm/nm) and DFS-12 (linear dispersion 1 mm/nm) equipped with FEU-100 and FEU-79 photomultipliers. The N_2_ laser (*λ*_ex_ = 337.1 nm), two diode-pumped lasers (*λ*_ex_ = 473 and 532 nm, respectively), and a xenon lamp (DKsSh-150) were used as sources of PL excitation. The PL spectra were studied as a function of the exciting radiation wavelength (*λ*_ex_) and were analyzed over a wide range of excitation and emission wavelengths (200–800 nm) and sample temperatures (77–300 K).

## Results and Discussion

### Morphology and Structure

**Fig. 1:**
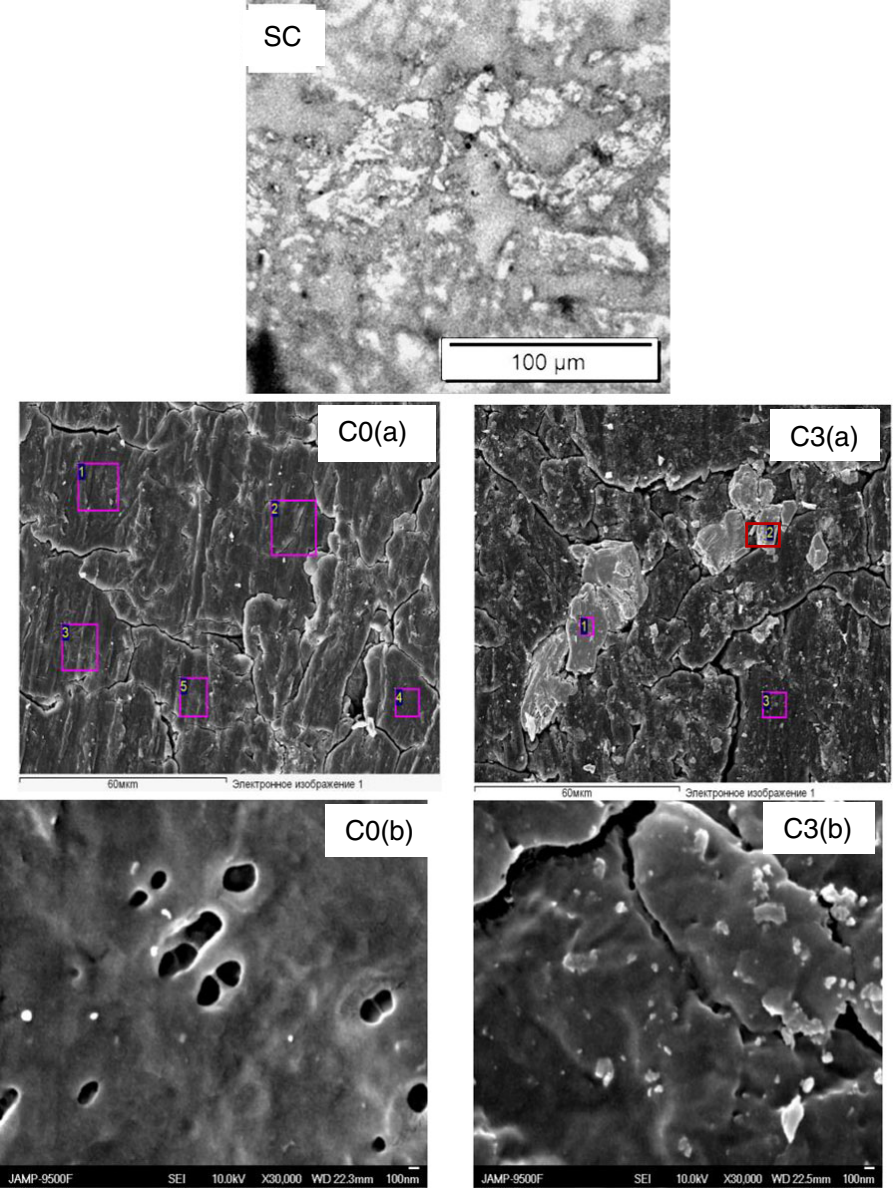
SEM images of the starting cellulose tablet (SC); dispersed and pressed micro/nanocellulose C0 sample (C0(**a**), C0(**b**)), cellulose-oxide micro/nanocomposite sample (C3(**a**), C3(**b**)). The image width is 120 μm for C0(**a**) and C3(**a**) and 3.6 μm for C0(**b**) and C3(**b**)

The surface of the all samples was monitored using a scanning electron microscope. The set of the obtained images of the various samples is available in Fig. 1. Figure 1SC is an image of a starting cellulose surface (sample SC). It is clearly seen that the sample consists of amorphous-phase areas and evenly distributed cellulose microcrystal conglomerates of predominant size near 5–40 μm. There are no clearly distinguished borders of microcrystals. Interfaces between them and the amorphous phase are blurred.

The un-doped M/NC C0 sample shows other morphology that consist of many closely located cellulose plates and their conglomerates of 5–20-μm sizes and overlapping or separated by faults/cracks (Fig. 1C0(a)). The craters/caverns can be found somewhere within an area of the plates (Fig. 1C0(b)).

The similar image is the characteristics for the C3 sample which contains a large amount of oxide component. The large cellulose grains and their conglomerates of 5–20-μm size are there. Besides, some inclusions in another view can be seen, and they reach 20-μm sizes (Fig. 1C3(a)). (The largest of them are marked by rectangles 1 and 2 in Fig. 1C3(a).) Their morphology can be described as clusters of the smaller size particles. There are a lot of such types of particles of smaller size, which are near 5–200 nm. It seems the particles lie on the surface of cellulose plates of 0.5–1-μm size, and the last ones can be separated with clearly seen faults/cracks (Fig. 1C3(b)).

Thus, we can state that morphology is significantly different for the starting microcellulose and the samples made by dispersion followed by pressure. Really, we can say that both the micro/nanocellulose samples (M/NC) and cellulose-oxide micro/nanocomposites (COM/NC) we made are “ceramics-like” materials. As we see below, the mentioned particles of small size are undoubtedly oxide compound inclusions in the cellulose host. Thus, we found that these inclusions effect cellulose host morphology and as for cellulose-oxide composites, the average sizes of cellulose grains are smaller. In any case, an assumption arose that prepared high-pressure composites are not a simple mechanical mixture of the cellulose host and oxide compounds, so this fact should be revealed in the physical properties of the composites.

#### Chemical Element Analysis

Chemical element analysis was performed for many specific zones of the samples using SEM microscope tools. Some of those zones are marked by colored rectangles and numbered in Fig. 1C0(a), C3(a). Of course, it was found that C and O are the predominant components of the un-doped C0 sample (see Table 1). Similar data was found for the C3 sample in zone 3. The sample C3 contains a compound oxide, but zone 3, however, is far from the abovementioned large particles. Only small particles are in this zone, and they contribute to the respective data of Table 1 as a low amount of K, Eu, and Mo. As for zones 1 and 2 of the C3 sample, chemical element analysis clearly indicates that their composition is close to the characteristic of K_2_Eu(PO_4_)(MoO_4_). Thus, large and small particles of the C3 samples described above are really inclusions of the oxide compounds.

**Table 1:** The content of some elements at the different zones of the C0 (in mas. %) and C1 samples (in at.%)

C0 sample	C	O				
Zone 1	63.89	36.11				
Zone 2	63.32	36.68				
Zone 3	64.88	35.12				
Zone 4	70.44	29.56				
Zone 5	67.75	32.25				
Average data	66.06	33.94				
C1 sample	C	O	K	Eu	P	Mo
Zone 1	9.28	60.92	9.78	5.74	6.51	7.77
Zone 2	4.21	62.51	9.07	7.93	7.31	8.96
Zone 3	75.61	24.00	0.01	0.11	0.00	0.27

#### XRD Analysis

**Fig. 2:**
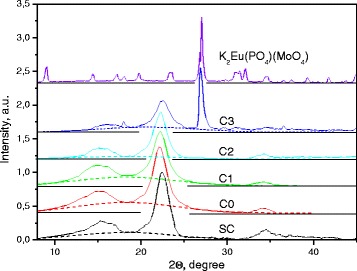
The XRD pattern of the starting microcellulose (*SC*), micro/nanocellulose (*C0*), cellulose-oxide micro/nanocomposites (COM/NC) with oxide concentration 0.2% (*C1*), 2.2% (*C2*), and 18.2% (*C3*) samples. The XRD pattern of the K_2_Eu(PO_4_)(MoO_4_) oxide is shown at the top of the Figure. The *dotted lines* are the Gauss curves approximating the contribution of the cellulose amorphous-phase into ﻿overall XRD scattering

The XRD pattern of the starting SC sample shows the set of diffraction peaks in the range 10°–45° of 2*θ* (Fig. 2, curve SC). There are some peaks of high intensity (these peaks are located at 15.9, 22.5, and 34.5° of 2*θ*). Some peaks were also registered as low-intensity features or shoulders (Fig. 2, curve SC).

Similar XRD results were obtained for the C0 micro/nanocellulose sample. As Fig. 2 (see curve C0) shows, intensive peaks as well as low-intensity features are located at the same peak positions as for the starting microcellulose sample. At the same time, detailed analysis has revealed that the full width at half maximum (FWHM) of the most intensive XRD peaks lying near 22.5° of 2*θ* slightly differs specially from the case of the starting non-dispersed microcrystalline cellulose: the SC sample. So, we can conclude that COM/NC samples such as those that had been treated by means of dispergation of the starting microcellulose are really characterized by other distribution of the cellulose crystallite sizes. The role of disordered interface layers of the crystallites can be higher too.

Manifestation of the additive peaks is a main peculiarity of the cellulose-oxide micro/nanocomposite (COM/NC) XRD patterns (Fig. 2, curves C1, C2, and C3). These peaks are already registered at near 27° and 56° of 2*θ* for the C2 sample (content of oxide is 2.2%), while there is a lot of oxide peaks in the ranges 30°–37° and 50°–62° of 2*θ* for the C3 sample (content of oxide is 18.2%). Intensive peaks are located near 13°, 27°, and 57° of 2*θ* (Fig. 2, curve C3). When oxide content increases, the relative intensity of the mentioned peaks increases also, while the relative intensity of the cellulose peaks decreases. Undoubtedly, the additive peaks caused by oxide components of composites and comparison with the XRD pattern of the K_2_(PO_4_)(MoO_4_) compound confirm that (Fig. 2).

Lattice periods calculated on the base of more intensive peak positions are given in Table 2. These data are typical for crystalline cellulose if compared with the published data (see, e.g., [25, 26] and elsewhere).

**Table 2:** Lattice periods (*d*_hkl_, nm) and crystallinity (*k*, %) for cellulose samples: starting microcellulose (SC), micro/nanocellulose (C0), cellulose-oxide micro/nanocomposites (COM/NC) with oxide concentration 0.2% (C1), 2.2% (C2), and 18.2% (C3)

Sample	*d* $1\overline{1}0$	*d*_200_	*d*_004_	*k*
SC	5.56	3.95	2.60	66
C0	5.85	4.02	2.62	56
C1	5.79	3.95	2.62	57
C2	5.72	3.98	2.61	58
C3	5.55	3.95	2.60	58

Cellulose crystallinity (*k*) was calculated for all studied samples. In order to perform the calculation, a contribution of the amorphous phase was extracted from the total XRD pattern. To do this, the last one was considered as a sum of the amorphous-phase background area, *I*_am_ (under bar lines with maximum position near 19° of 2*θ*, Fig. 2), and the area of peaks related to the cellulose crystal phase, *I*_cr_. These peaks are located in the background. When we deal with calculations for the C2 and C3 samples, a contribution of oxide peaks was extracted previously. So, crystallinity level was evaluated as a ratio:

$$ \kappa =\frac{I_{\mathrm{cr}}}{I_{\mathrm{cr}}+{I}_{\mathrm{am}}} $$

Calculated *k* values for the all studied samples are also given in Table 2.

Thus, we can summarize that treatment and oxide doping influence micro/nanocellulose cellulose characteristics (lattice periods, crystallinity, size of crystals), but detailed study is needed in the future to clarify the mechanisms of this influence.

The mechanical and thermal properties of prepared cellulose composite samples studied in the work are discussed below.

#### Density

First, it should be noted that the density of all the samples was calculated. In spite of the simplicity of this physical property, density can be very useful for clarifying a material’s structure, especially when composite materials are under study. For example, density data could be important when the question arises about additivity of composite material density. As calculated by us, the average values are 1.241, 1.260, 1.153, 1.147, and 1.284 g/cm^3^ for the SC, C0, C1, C2, and C3 samples, respectively.

### TGA and DSC

**Fig. 3:**
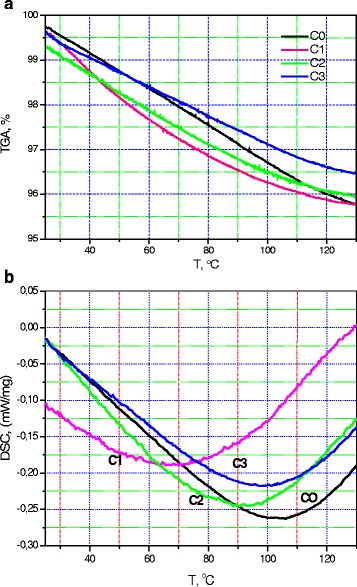
Thermogravimetry (**a**) and differential scanning calorimetry curves (**b**) for the cellulose-oxide micro/nanocomposite: un-doped sample (*C0*), samples with oxide content 0.22% (*C1*), 2.2% (*C2*), and 18.2% (*C3*)

The thermogravimetric analysis (TGA) curves for the un-doped CM/N, C0, and C1 samples intersect at 25 and 125 °C, while in the whole temperature range between these points, 25–125 °C, the C0 sample curve is higher than the C1 curve (Fig. 3a). The behavior of these curves is different: the first of them is convex and the second is concave. So, the difference between them is largest in the temperature range near 60–80 °C. The TGA curves for the C2 and C3 samples show similar temperature behavior, but the difference between them increases when temperature increases. In fact, the difference is near 0.3% at 25 °C, and it is near 0.6% when the temperature rises up to 125 °C. Thus, we can note that the difference between the dependency for the C0 sample, which does not contain an oxide component, and the C1 sample, where the oxide amount is minimal, is appreciable. The dependencies for the C2 and C1 samples are close, while characteristics of the C3 sample differ considerably from the C2 sample, in spite of the similar difference in the oxide contents: about ten times in both cases.

The differential scanning calorimetry (DSC) curves (Fig. 3b) have demonstrated similar behavior for the C0, C2, and C3 samples up to a temperature near 70 °C, when difference have arisen between them. As a result, calorimetric singularity is near 103, 92, and 98 °C for the C0, C2, and C3 samples, respectively. A big difference is observed between the DSC curve for the C1 sample and the other DSC curves. A mentioned fact concerns both of the curves’ shape and singularity positions. The last one is near 67 °C for the C1 sample.

Thus, we can note that considerable difference between characteristics of the C0 sample, which does not contain oxide component, and the C1 sample is obvious. This difference can be regarded as predicable, as it reflects radical difference in their composition. At the same time, noticeable similarity of the C0 and C3 sample properties is unexpected and has to be an object of future studies. The prepared composite heat capacity will also be a subject of our investigation as these data are important for possible practical applications.

**Fig. 4:**
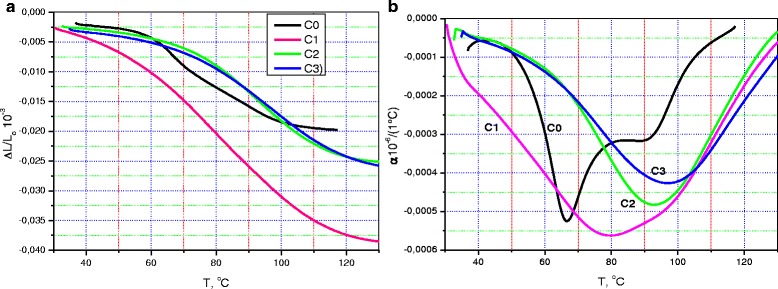
**a** Temperature dependencies of the relative change in length, Δ*L*/*L*(*T*), and **b** linear coefficient of thermal expansion, *α*(*T*), measured along the height of the disc-like C0, C1, C2, and C3 pristine samples (before heating)

**Fig. 5:**
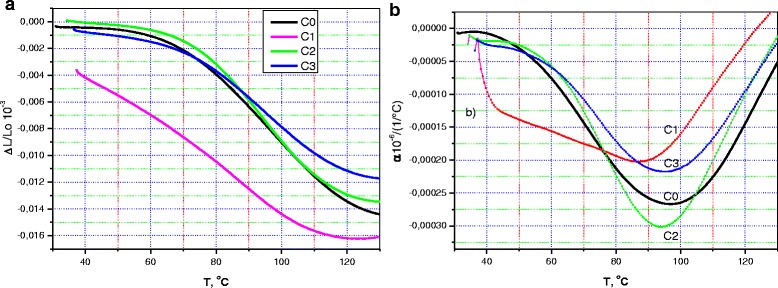
**a** Temperature dependencies of the relative change in length, Δ*L*/*L*(*T*), and **b** linear coefficient of thermal expansion, *α*(*T*), measured along the height of the disc-like C0, C1, C2, and C3 samples after their preheating

Figure 4a, b illustrate the dilatometric behavior of the samples under study. It should be noted that the measurements were made twice. First, the dilatometry studies were performed before the TG analysis, as some effect of heating on the sample composition was predicted. Thus, Fig. 4a shows the relative variation in length, Δ*L*/*L*, of the pristine CM/N sample, C0, and of the cellulose-oxide micro/nanocomposites, COM/NC, containing 0.2, 2.2, and 18.2% oxide components. Figure 5a, b demonstrate dilatometric behavior of the same samples but taken after TGA. The measurements were made in both cases along the height of the discs.

In discussing the results of dilatometric behavior of the Δ*L*/*L* dependencies for the pristine samples, it is easy to observe similar variation in dimension for all the COM/NC samples, while the Δ*L*/*L* behavior of the C0 sample differs radically. At the same time, Δ*L*/*L* variations are in the same range for C0, C2, and C3 samples, despite the different behavior of the corresponding Δ*L*/*L* curves. This range is near 13–23 × 10^−3^, while Δ*L*/*L* reaches a value of 40 × 10^−3^ for the C1 sample with a minimal amount of oxide component.

The description made above coincides with the data about coefficient of thermal expansions (Fig. 4b). We see that the coefficient of thermal expansion, *α*(*T*), varies differently with temperature and the difference in variation is significant for the C0 sample and C1, C2, and C3 samples. The hidden peak of the *α*(*T*) curve is below 40 °C and two strong dilatometric singularities are near 67 and 90 °C for the C0 sample. Similar three features form the *α*(*T*) curve for the C1 sample, we suppose, but they are smeared and shifted to the high-temperature side, if compared with the C0 sample. (Their positions are 35, 80, and 100 °C). When the oxide amount increases, only one singularity is near 92 and 97 °C for the C2 and C3 sample *α*(*T*) curves, respectively. Described peculiarities imply that the samples are strongly different. Indeed, the first of them is a “pure” micro/nanocellulose, and other ones are “cellulose-oxide” composite materials. However, this explanation is quite simplified, and dilatometry data taken after the thermal effect (it resulted from the performed TGA procedure) confirm this view.

We see from Fig. 5a, b that the dilatometric behavior of Δ*L*/*L* for the un-doped C0 sample was changed after heating and became like the dependencies described above for the other oxide-containing samples (Fig. 5a). Similarly, the coefficient of thermal expansion for the C0 sample was changed too that had resulted in similarity of the *α*(*T*) curves for the C0, C2, and C3 samples (Fig. 5b). At the same time, dilatometric characteristics and their temperature dependencies for the C1 sample appreciably differs from other dependencies. However, the C1 curve can be described as a superposition of the typical for all curves with a wide high-temperature peak near 95–97 °C and the abovementioned low-temperature singularities (see Figs. 4b and 5b, C1 curve). It is interesting to analyze the behavior of the high-temperature peak intensity (comparing the data in Figs. 4b and 5b). These figures shows that, only for C0 after heating, the dilatometric peak retains its intensity (about 250–300 1/°C), while for all other samples, the intensity of this peak decreases of 1.5–2 times (560, 480, 420 1/°C before preheating and 200, 300, 230 1/°C after preheating for the C1, C2, C3 samples, respectively). Besides, heating leads to decrease of the low-temperature (30–60 °C) side of all the *α*(*T*) curves. Thus, we can state that several various mechanisms determine the thermal expansion for both un-doped micro/nanocellulose and cellulose-oxide micro/nanocomposite materials. Right now, there are not enough data to discuss those mechanisms, but we can suppose that the kinetics of the cellulose particle prevails on the whole temperature range, where measurements have been made. Besides, some resorption of ambient gases and change of the sample humidity can influence the obtained data. (See also the TGA and DSC results described above). In any case, it should be emphasized that addition of a small amount of oxide compound (near 0.2%) decreased the coefficient of thermal expansion of the micro/nanocellulose samples and this fact corresponds to the increase of the interaction forces within the materials. The value of the relative change in the length of the un-doped sample also decreases twice when 0.2% oxide had been added and the C1 sample was heated (Fig. 4a). The abovenoted results can be of importance for applications of similar cellulose-oxide micro/nanocomposites in advanced mechanical and thermomechanical devices.

### Dielectric Properties

**Fig. 6:**
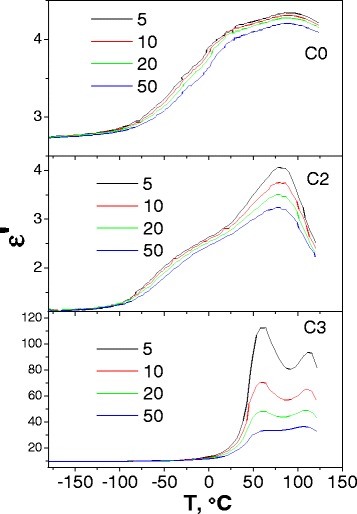
Temperature dependencies of the complex dielectric permittivity real part, *ε*′, for C0, C2, and C3 pristine samples measured at the frequencies *f* = 5, 10, 20, and 50 kHz

The real part of the complex dielectric permittivity was measured in the temperature range −180 to 130 °C (Fig. 6). As Fig. 6 shows, a small oxide amount already leads to some changes in *ε*′(*T*) dependencies and these changes are larger for higher frequency curves, so the curves corresponding to various frequencies are diverged if compared with Fig. 6a, b. The *ε*′ values decreasing in the low temperature range, −180 to 50 °C, is observed too; thus, the wide peak near 75 °C becomes distinguished. If the amount of oxide increases, this peak splits into two ones located near 62° and 93°. Simultaneously, the abovementioned low-temperature range of the *ε*′ practically vanishes in the range −180 to 0 °C.

**Fig. 7:**
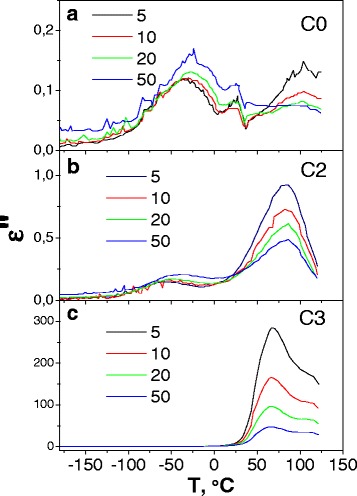
Temperature dependencies of the complex dielectric permittivity imaginary part, *ε*″, for C0 (**a**), C2 (**b**), and C3 (**c**) pristine samples, measured at the frequencies *f* = 5, 10, 20, and 50 kHz

The imaginary part of the complex dielectric permittivity measured in the same temperature range −180 to 130 °C is shown in Fig. 7. Figure 7 shows two peaks near −50 and 92 °C for the C0 and C2 samples, respectively. The first of them is shifted to the high-temperature side if the temperature increases. As for the second peak, its intensity decreases if the frequency increases. Therefore, the first peak should be related to the dielectric relaxation polarization, while the second can be related to the phase transition evaporation of water.

A dielectric relaxation is observed for real and imaginary parts of the dielectric permittivity for the C0 and C2 samples near −50 °C caused by reorientation of the cellulose methyl groups on the micro/nanocellulose crystallite surface via changing their conformation from tg toward tt. At a higher oxide concentration (C3 sample), dielectric relaxation disappeared (see *ε*′(*T*) and *ε*″(*T*) dependencies in Fig. 7). We can conclude that oxide particles block methyl groups’ conformation movement on the cellulose micro/nanocrystallite surface.

### Luminescence Properties

**Fig. 8:**
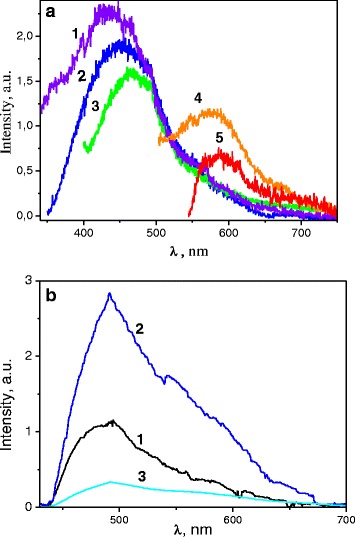
The PL spectra of the SC/C0 samples: **a** pristine samples excited under various excitation wavelengths: *λ*_ex_ = 300 nm (*1*), 337.1 nm (*2*), 370.5 nm (*3*), 473 nm (4), and 532 nm (*5*) [11]; **b** “before” (*1*) and after thermal treatment at *T*_tr_ = 200 °C (*2*) and 273 °C (3). *λ*_ex_ = 405 nm; *T* = 23 °C

All the studied samples are characterized with intensive photoluminescence (PL). The composed PL band consists of at least three components lying in the 325–750-nm range (Fig. 8, [11]). The PL had been observed both in the PL spectra of the starting microcellulose, SC, and the un-doped C0 samples under excitation in the wide range of excitation wavelengths, *λ*_ex_, from 300 up to 550 nm. The shape, peak position, *λ*_max_, and intensity of the PL band depend on the *λ*_ex_ that had been found by us previously for the similar samples [10, 11]. The spectra and their dependencies on the *λ*_ex_ are similar for the mentioned samples: *λ*_ex_ increases as the peak position, *λ*_max_, reveals a tendency to the long-wavelength-side shifting. Mainly, the component with a peak position near 430–470 nm dominates in the spectra at UV excitation, while at the visible laser power excitation (*λ*_ex_ = 405–532 nm), an additional low-intensity PL band appears. The band is located at longer wavelengths, range 500–700 nm, than that of the UV excitation. The peak position of the band, *λ*_max_, is near 680–590 nm (Fig. 8a) [11].

**Fig. 9:**
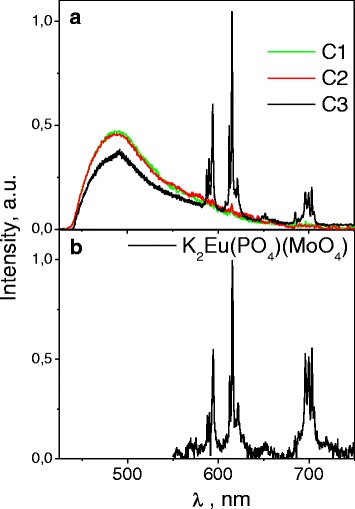
The PL spectra of the C1, C2, and C3 pristine samples (*λ*_ex_ = 405 nm) (**a**) and the PL spectra of the K_2_Eu(MoO_4_)(PO_4_) free powder (*λ*_ex_ = 405 nm; *T* = 23 °C) (**b**)

When the SC and C0 samples underwent the thermal treatment, the PL spectra changed but these changes are small if the treatment temperature, *T*_tr_, is lower than 230 °C (Fig. 9). Only some decrease of the short-wavelength side of the spectra takes place. At the same time, the PL intensity changes and this change is not monotonous: intensity increases when *T*_tr_ increases up to 200 °C, then the PL intensity rapidly decreases (Fig. 9b).

The luminescence peculiarities of the cellulose samples described above are similar to those reported earlier (see, e.g., [27]). The luminescent findings presented here proved that there is more than one luminescence center in the studied cellulose samples. The multicomponent structure of the PL bands is usually related to the manifestation of several types of organic chromophores, and those are in the host cellulose. Carbonyl groups and different kinds of low-molecular derivatives of cellulose destruction are between them. The nonmonotonous behavior of the host cellulose PL intensity observed by us can be related with the resorption of some ambient atmosphere components and particularly with water removal. The mentioned factor has to lead to luminescence enhancement when the treatment temperature increases up to 200 °C. If treatment temperature is higher than 200 °C, structural changes of the micro/nanocellulose host occur that leads to luminescence quenching.

Taking into account the abovementioned items, we understand that incorporation of oxide compounds must change the PL properties of the micro/nanocellulose host (Fig. 9). The PL spectra of the C1, C2, and C3 composites are shown in Fig. 9.

It is seen that if the amount of oxide is low (C1 sample), the PL of the host cellulose prevails in the spectra. In fact, the C1 spectrum is very similar to the spectra shown in Fig. 8b. Nevertheless, the low-intensity PL lines can be seen in the spectral range 550–700 nm both for the C1 and C2 samples. When the oxide concentration is higher (C3 sample), significant changes are observed: narrow lines become more intensive than host micro/nanocellulose luminescence, and moreover, the cellulose PL intensity decreases. No doubt the additive PL lines are related with luminescence processes in the oxide component of the composites. A comparison of described spectra with the PL spectra of the free powder K_2_Eu(MoO_4_)(PO_4_) compounds confirms this statement (Fig. 9). So, the PL lines are ascribed to the ^5^D_0_→^7^F_J_ (*J* = 1 ÷ 4) radiation transitions in the inner *f-f* electronic shells of the Eu^3+^ ions which are in the composition of the oxide compound. Quenching of the cellulose host PL, when the amount of the oxide component increases, can be caused by the destroying effect of the oxide micro/nanoparticles on the morphology and structure of the COM/NC material, as we already have pointed out above. So, the PL characteristic behavior also indicates an interaction between the cellulose host and oxide component of the composite material.

## Conclusions

Composite materials that consist of micro/nanocellulose and complex K_2_Eu(MoO_4_)(PO_4_) luminescent oxide particles were made using mechanical dispersion and followed high pressing (1.8 × 10^4^ kPa/m^2^).

The structure of the composite without oxide is formed by grains of nearly 5–50 μm in size (crystallinity is about ~56%), which allowed us to characterize the made samples as ceramics-like. Structure of the micro/nanocellulose samples which contain an oxide particle is similar, but the cellulose grains are deformed by oxide particles. The sizes of the last ones are from ~10 nm up to 10 μm.

Physical properties of studied materials (density, crystallinity, relative extension, thermal extension coefficient, dielectric relaxation parameters, spectra, and intensity of photoluminescence) depend both on the sample temperature (conditions of the thermal treatment) in the range 25–125 °C and content of oxide component.

It was assumed that the difference in oxide particle size distribution for the samples of various oxide concentration and molecules of water (~0.3–0.5%) determine the complexity of the abovementioned property dependencies on temperature and oxide content.

Obtained characteristics show that similar composite materials are promising to be used for the creation of advanced mechanical, thermomechanical, electromechanical, and optoelectronic devices, particularly for white emitting diode creation.

## Abbreviations

COM/NC: Cellulose-oxide micro/nanocomposite
FWHM: Full width at half maximum
M/NC: Micro/nanocrystalline cellulose
PL: Photoluminescence
SEM: Scanning electron microscopy
XRD: X-ray diffraction
